# Prediction of short-term progression of COVID-19 pneumonia based on chest CT artificial intelligence: during the Omicron epidemic

**DOI:** 10.1186/s12879-024-09504-9

**Published:** 2024-06-17

**Authors:** Xinjing Lou, Chen Gao, Linyu Wu, Ting Wu, Linyang He, Jiahao Shen, Meiqi Hua, Maosheng Xu

**Affiliations:** 1 Department of Radiology, The First Affiliated Hospital of Zhejiang Chinese Medical University (Zhejiang Provincial Hospital of Chinese Medicine), 54 Youdian Road, Hangzhou, Zhejiang 310006 China; 2 The First School of Clinical Medicine, Zhejiang Chinese Medical University, 548 Binwen Road, Hangzhou, Zhejiang 310053 China; 3 Hangzhou Jianpei Technology Company Ltd. Xiaoshan District, Hangzhou, Zhejiang 311200 China

**Keywords:** COVID-19, SARS-CoV-2, Computed tomography, Risk factors, Prognosis analysis

## Abstract

**Background and purpose:**

The persistent progression of pneumonia is a critical determinant of adverse outcomes in patients afflicted with COVID-19. This study aimed to predict personalized COVID-19 pneumonia progression between the duration of two weeks and 1 month after admission by integrating radiological and clinical features.

**Methods:**

A retrospective analysis, approved by the Institutional Review Board, encompassed patients diagnosed with COVID-19 pneumonia between December 2022 and February 2023. The cohort was divided into training and validation groups in a 7:3 ratio. A trained multi-task U-Net network was deployed to segment COVID-19 pneumonia and lung regions in CT images, from which quantitative features were extracted. The eXtreme Gradient Boosting (XGBoost) algorithm was employed to construct a radiological model. A clinical model was constructed by LASSO method and stepwise regression analysis, followed by the subsequent construction of the combined model. Model performance was assessed using ROC and decision curve analysis (DCA), while Shapley’s Additive interpretation (SHAP) illustrated the importance of CT features.

**Results:**

A total of 214 patients were recruited in our study. Four clinical characteristics and four CT features were identified as pivotal components for constructing the clinical and radiological models. The final four clinical characteristics were incorporated as well as the RS_radiological model to construct the combined prediction model. SHAP analysis revealed that CT score difference exerted the most significant influence on the predictive performance of the radiological model. The training group’s radiological, clinical, and combined models exhibited AUC values of 0.89, 0.72, and 0.92, respectively. Correspondingly, in the validation group, these values were observed to be 0.75, 0.72, and 0.81. The DCA curve showed that the combined model exhibited greater clinical utility than the clinical or radiological models.

**Conclusion:**

Our novel combined model, fusing quantitative CT features with clinical characteristics, demonstrated effective prediction of COVID-19 pneumonia progression from 2 weeks to 1 month after admission. This comprehensive model can potentially serve as a valuable tool for clinicians to develop personalized treatment strategies and improve patient outcomes.

**Supplementary Information:**

The online version contains supplementary material available at 10.1186/s12879-024-09504-9.

## Introduction

As of August 2023, more than 769 million confirmed Corona Virus Disease 2019 (COVID-19) cases have been reported worldwide, and more than 6.9 million deaths have occurred [1]. Severe acute respiratory syndrome coronavirus 2 (SARS-CoV-2) has been demonstrated to be more transmissible than other coronaviruses, resulting in higher case numbers and the global pandemic. The B.1.1.529 (Omicron) variant of SARS-CoV-2, the virus that causes COVID-19, was first clinically identified in the United States in December 2021, and had become the predominant strain by late December. The omicron variant is more transmissible and less virulent than previously circulating variants [2]. Despite the containment of the current outbreak, there remains a potential for future recurrences. Therefore, we must enhance our understanding and establish standardized response mechanisms to manage potential health hazards effectively.

Persistent lung infection is a risk factor for poor prognosis; as some COVID-19 patients progressed from severe pneumonia to pulmonary edema, acute respiratory distress syndrome, multi-organ failure, and death [3]. Most current studies have focused on the clinical prognosis and the long-term pulmonary sequelae following infection of COVID-19 patients. However, the short-term progression of the lesions also needs attention to help better manage patients and adjust treatment regimens [4, 5].

Chest computed tomography (CT) has played a vital role as a diagnostic method, with a sensitivity of 67–100% and a specificity of 25–80% in diagnosing COVID-19 pneumonia patients [6–8]. Furthermore, chest CT is valuable for monitoring the severity and progression of COVID-19 pneumonia. In the context of the COVID-19 pandemic, a growing body of research has explored the potential of combining artificial intelligence (AI) with chest CT scans to predict treatment outcomes and prognosis [9]. The quantitative analysis of chest CT greatly influences the management of COVID-19. It has been used for severity of illness, hospitalization rates, intensive care unit (ICU) admissions, and mortality assessment [10–12]. However, the manual segmentation of lesions before the extraction of CT quantitative features is time-consuming and laborious. Automatic segmentation of chest CT lesions using deep learning technology increases the clinical applicability for quantitative CT evaluation of COVID-19 pneumonia.

In this study, we used the trained chest CT-based Multi-task U-Net model to segment COVID-19-related lung lesions. A large number of quantitative features were then extracted and combined with clinical characteristics to generate a combined clinical-radiological model for predicting the radiological prognosis of COVID-19 pneumonia after two weeks.

## Materials and methods

### Study population

The retrospective study was approved by the ethics committee of the First Affiliated Hospital of Zhejiang Chinese Medical University (Zhejiang Provincial Hospital of Chinese Medicine), and written informed consent was waived. Patients diagnosed with COVID-19 pneumonia in our hospital were retrospectively collected between December 2022 and February 2023. All patients underwent multiple chest CT scans upon admission and throughout their hospitalization. The inclusion criteria were: (1) positive SARS-CoV-2 RT-PCR or rapid antigen test results; (2) the interval of chest CT follow-up should be at least 14 days. The exclusion criteria were as follows: (1) age < 18 years; (2) non-emerging inflammatory conditions; (3) a history of pulmonary malignancy, lobectomy, or tuberculosis; or (4) co-infection with other bacteria or viruses.

### Clinical data collection

The clinical and laboratory information were collected from the electronic medical records, including baseline age, gender, medical history, signs and symptoms, c-reactive protein (CRP), leukocyte, lymphocyte count, lymphocyte percentage, neutrophil count, neutrophil percentage, procalcitonin, erythrocyte sedimentation rate (ESR), d-dimer, aspartate aminotransferase (AST), ferritin, lactic dehydrogenase (LDH), troponin I (TNI), N-terminal prohormone of brain natriuretic peptide (NT-proBNP) and interleukin-6 (IL-6). According to the “Diagnosis and Treatment Protocol for Novel Coronavirus Pneumonia (Trial Version 7)”, patients are classified as non-serious (moderate) and serious (severe, critical) [13, 14]. Clinical progression was defined as the deterioration of the initial clinical classification during follow-up, such as going from moderate to severe or severe to critical, as defined by the same protocol.

### CT acquisition

Patients underwent non-enhanced CT scans in the inspiratory breath-hold supine position with either low-dose or routine-dose CT scans. The scope of the scan includes the thoracic inlet to the costophrenic Angle. All CT images were acquired by one of four multi-slice CT scanners (TOSHIBA Aquilion ONE, SIEMENS Somatom Sensation 64, UIH uCT 530, or Siemens Somatom Definition AS). The CT scan parameters were as follows: tube voltage 120 kV; automatic tube current modulation; collimation 0.6 mm × 64 (Siemens), 0.5 mm × 64 (Toshiba), and 0.55 mm ×40 (UIH); matrix 512 × 512; slice thickness 1.0 mm (Siemens, Toshiba and UIH); reconstructed convolution function B40f (Siemens), lung smooth (Toshiba), and B sharp C (UIH).

### CT image interpretation

Short-term persistent progression of pneumonia was radiologically defined as any of the following: Persistent progression or recurrence of pneumonia two weeks to 1 month after baseline chest CT image follow-up [15, 16]. According to the radiologically progression of COVID-19 pneumonia, the patients were divided into the training and validation groups by stratified sampling at a ratio of 7:3.

The following CT findings were assessed and recorded: distribution of pneumonia preferences (bronchovesicular distribution, subpleural and diffuse), lobular septal thickening, pleural effusion, lymph node enlargement, emphysema, bronchiectasis, and pericardial effusion. For the radiological assessment, two experienced chest radiologists reviewed the images independently and concluded by consensus when disagreements arose. Following the chest CT scoring (CCTS) system proposed by Pan et al., each lobe’s degree of involvement was categorized into six grades: 0%, < 5%, 5–25%, 26–49%, 50–75%, and > 75%. These percentage ranges were given numerical scores from 0 to 5, respectively. The total chest CT score was then computed by summing the individual scores assigned to each lung lobe, ranging from 0 to 25 [15, 17]. In this study, the CCTS was evaluated accurately using these quantitative CT features. The peak CT score was defined as the CT score at the time of the most severe lung involvement, and the CT score difference was defined as the difference between the peak CT score and the initial CT score. All images were independently reviewed by two thoracic radiologists (with 4 and 8 years of diagnostic experience, respectively) and any disagreements were resolved by consensus.

### Image segmentation and quantitative feature extraction

**Fig. 1:**
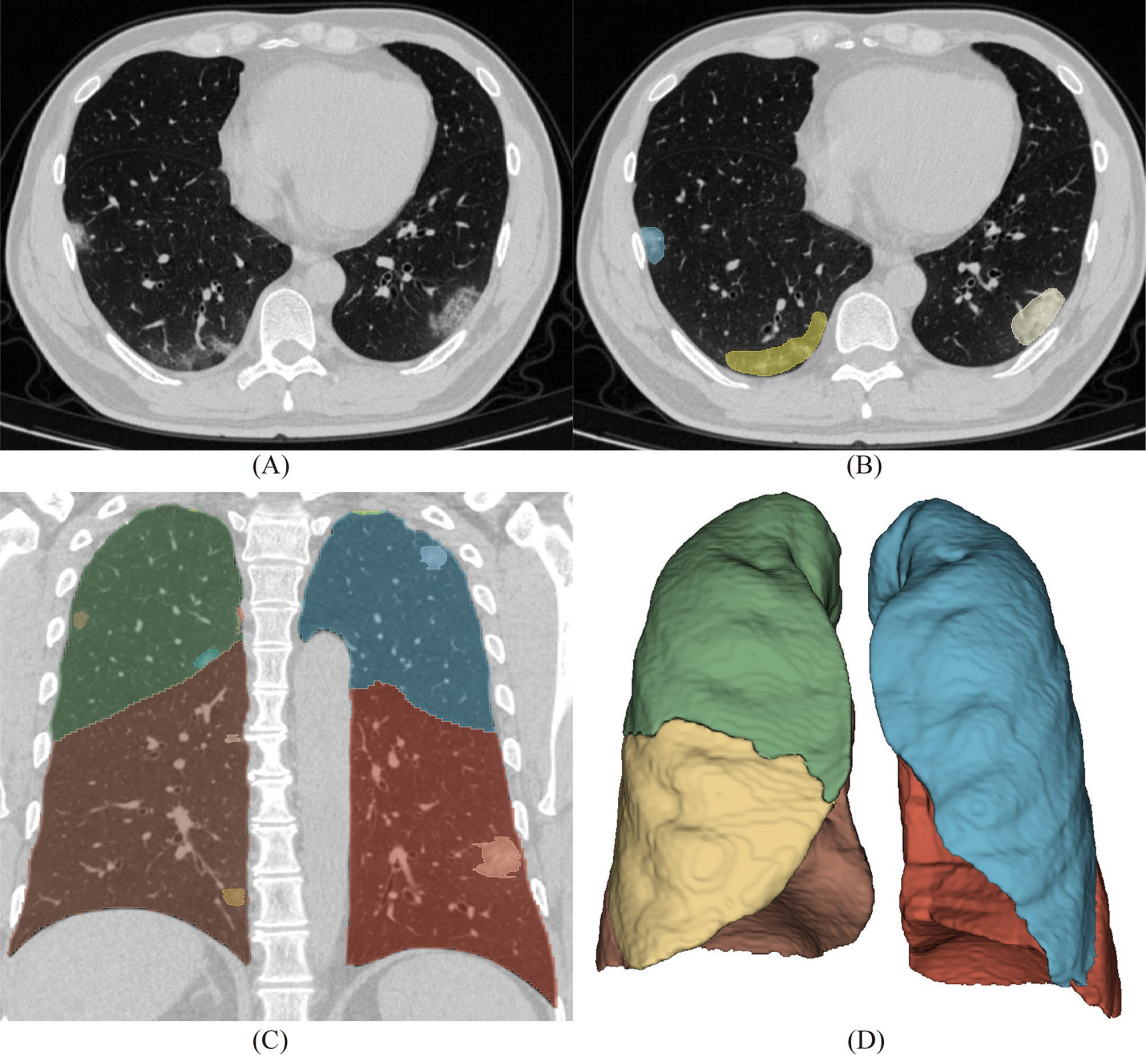
Illustration of imaging segmentation. **(A)** a CT image of a COVID-19 patient; **(B)** U-Net-based lesion segmentation on CT images; (**C**, **D**) Coronal and reconstructed CT images of lung lobe segmentation

The segmentation of COVID-19 pneumonia on CT images was performed using a trained multi-task U-net network (Fig. 1). A previous study has demonstrated that this deep learning model can accurately segment lung and COVID-19 on CT images, with an average Dice coefficient of 0.864 [18]. With its end-to-end learning capabilities, the multi-task U-Net network effectively utilizes features extracted from different tasks through simultaneous multi-task training. This approach enhances the model’s accuracy, accelerates its learning rate, and improves its interpretability. Quantitative features were extracted based on the mask of the lung and COVID-19 pneumonia obtained by automatic segmentation. These features included the volume and density of the whole lung and each lung lobe, the number of lesions, the volume and density of the lesions, and the ratio of the lesion volume to the total lung volume. The segmentation network in this study is implemented based on the Dr. Pecker cloud platform (http://www.jianpeicn.com/category/yuepianjiqiren).

### Feature selection and models construction

The eXtreme Gradient Boosting (XGBoost) algorithm was employed for model construction using the selected CT features after dimensionality reduction, including both CT visual features and quantitative CT features.

**Fig. 2:**
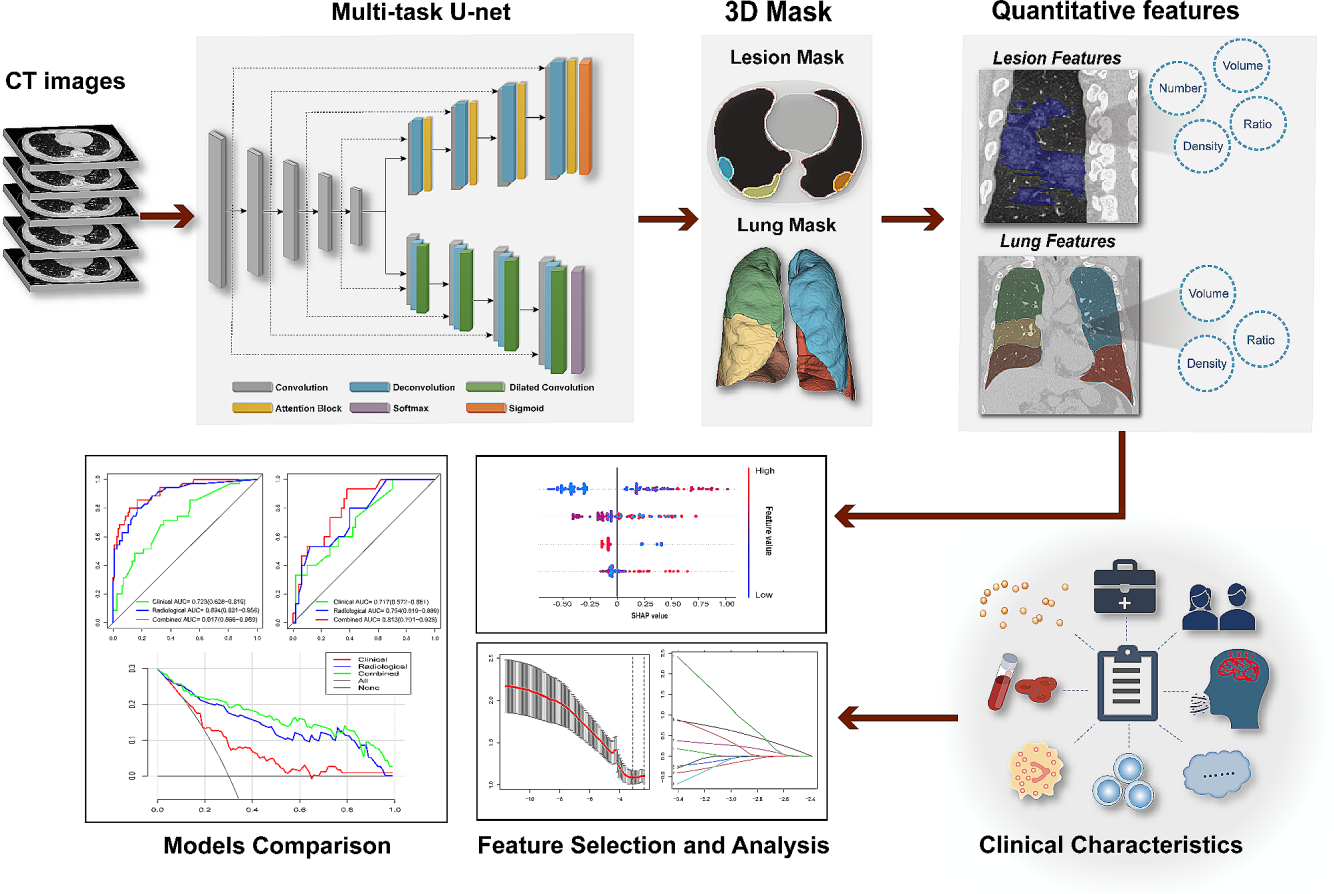
The overall workflow of this study

The Least Absolute Shrinkage and Selection Operator (LASSO) was initially employed to select clinical characteristics. A stepwise regression based on the Akaike Information Criterion (AIC) was then performed to identify significant features and establish a clinical model. A multivariate logistic regression analysis was conducted to establish a combined model that integrates the selected CT features with the chosen clinical characteristics. The overall flow chart is shown in Fig. 2.

### Statistical analysis

Continuous variables were expressed as mean and standard deviation (SD), and group comparisons were made using the t-test. Categorical variables were represented as counts and percentages, with the appropriate statistical tests (Chi-square or Fisher’s exact tests) employed for between-group comparisons. The predictive performance of the established models was assessed using the area under the Receiver Operating Characteristic (ROC) analysis. The Hosmer-Lemeshow test was employed to determine the model’s goodness of fit. Additionally, Decision Curve Analysis (DCA) was carried out to ascertain the clinical utility of the model. The Shapley Additive explanations (SHAP) method was used to illustrate the importance of CT features and their impact on the overall predictive model. All analyses were conducted with R software version 4.3.0. A two-tailed P-value of less than 0.05 was determined to denote statistical significance.

## Results

### Clinical characteristics

**Fig. 3:**
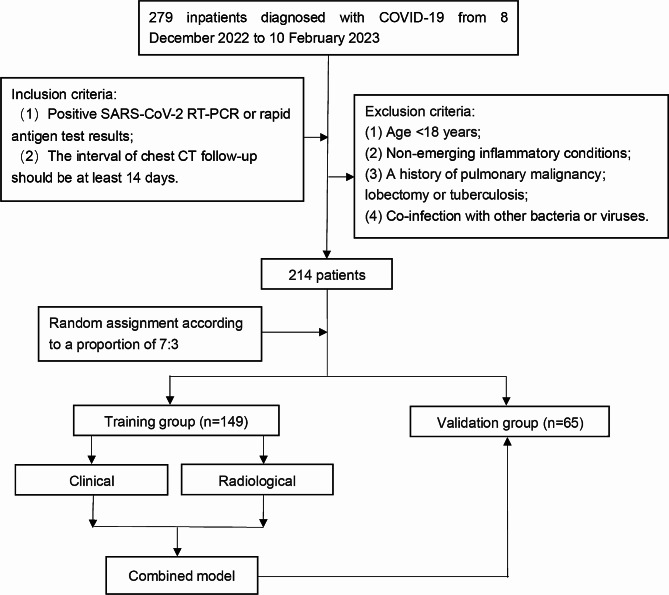
Flowchart of the patient selection process

The study finally included two hundred fourteen patients with COVID-19 pneumonia (Fig. 3). There were no significant differences in clinical characteristics between the training and validation groups (all *P* > 0.05). The clinical characteristics of the training and validation groups were detailed in supplementary material.

### Feature selection and models construction

**Fig. 4:**
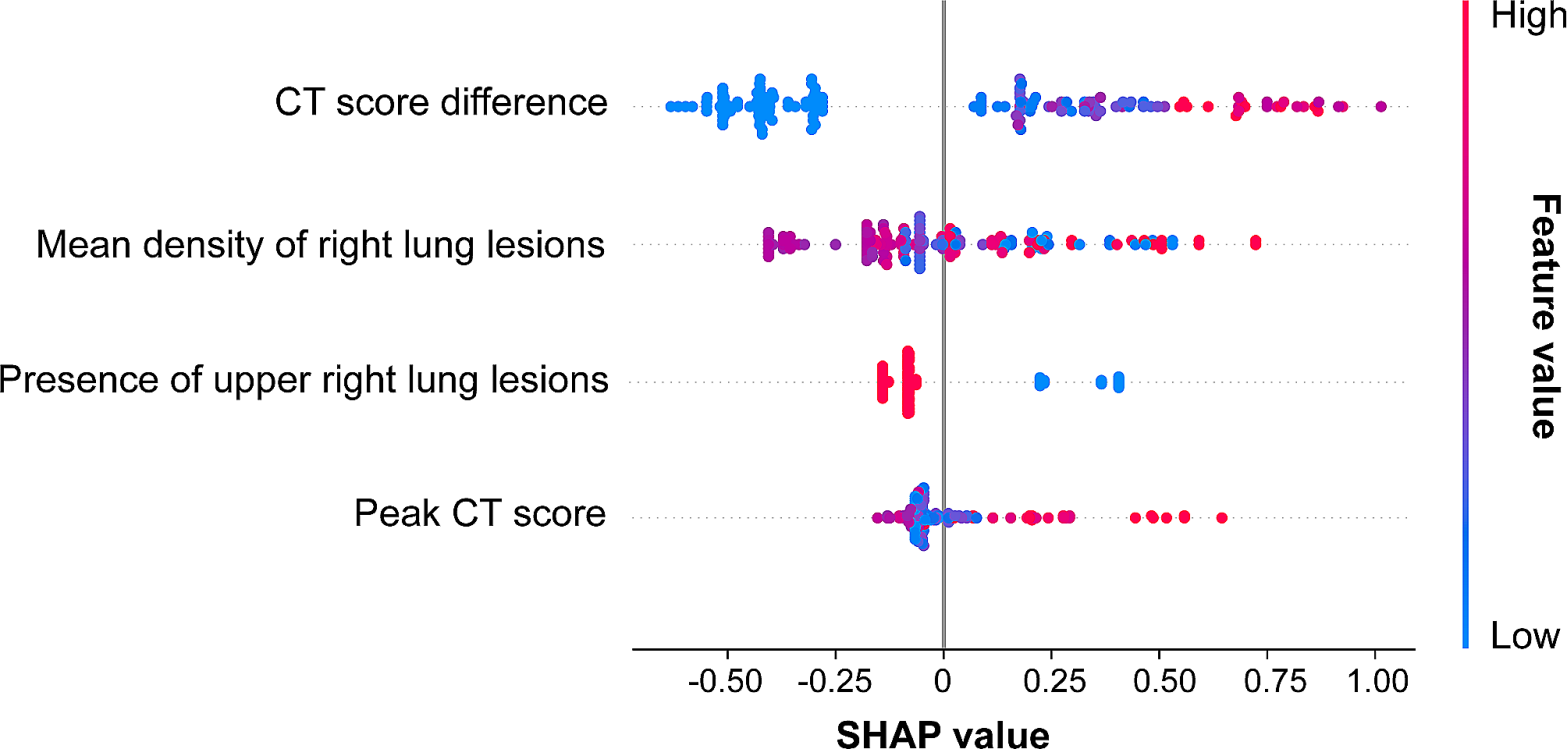
SHAP summary plots of the radiological model. The diagram illustrates the feature attributions to the model’s predictive performance. Blue indicates low eigenvalues, and red indicates high eigenvalues

CT features were downscaled and modelled using the Xgboost method to obtain the final risk scores RS_radiological model. The CT features were sorted according to their global importance. The top 4 features after dimension reduction by XGBoost to build the radiological model were: CT score difference, mean density of right lung lesions, presence of upper right lung lesions, and peak CT score. The SHAP summary plot showed that the continuous progression of pneumonia was positively correlated with the CT score difference and peak CT score and negatively associated with the presence of upper right lung lesions (Fig. 4). However, the mean density of right lung lesions may exhibit a complex relationship with the model’s output.

After initial inclusion of 32 clinical characteristics, dimensionality reduction was performed using the LASSO method, resulting in 8 remaining features: NT-proBNP, hypertension, ESR, diarrhea, cough, clinical progression, chronic diseases, and dyspnea. Subsequently, a stepwise regression method further refined the model to include the final four clinical characteristics: NT-proBNP, ESR, clinical progression, and dyspnea. After stepwise regression we integrated the radiological and clinical models to establish a combined model encompassing all individual models’ features. The features and corresponding coefficients used to construct the clinical model and the combined model are shown in Tables 1 and 2. The results of the Hosmer-Lemeshow test indicated that all models exhibited no evidence of overfitting (all *P* > 0.05). The risk scores (RS) for each patient’s clinical and combined models were calculated using the following formula:

**Table 1:** Stepwise regression coefficients for the clinical model

Variable	Estimate	StdError	t.value	*P*.value
Clinical Progress	0.307789	0.085774	3.588383	0.000456
Hypertension	-0.090615	0.057932	-1.564172	0.119988
Diarrhea	-0.219267	0.122708	-1.786902	0.076072
Dyspnea	0.136200	0.067313	2.023401	0.044895
ESR	0.616147	0.171404	3.594711	0.000446
NT-ProBNP	1.104669	0.415625	2.657852	0.008759

**Table 2:** Stepwise regression coefficients for the combined model

Variable	Estimate	StdError	t.value	*P*.value
Clinical Progress	0.270233	0.073071	3.698224	3.080474e-04
Dyspnea	0.145534	0.058518	2.486991	1.402448e-02
ESR	1.069441	0.148656	7.194072	3.175357e-11
NT-ProBNP	0.843065	0.344778	2.445238	1.568233e-02
RS_Rad	0.258173	0.035708	7.230036	2.615308e-11

$$\begin{gathered}RS\_clinical{\text{ }}model = Clinical{\text{ }}Progress*1.439040162417479 \hfill \\\,\,\,\,\,\,\,\,\,\,\,\,\,\,\,\,\,\,\,\,\,\,\,\,\,\,\,\,\,\,\,\,\,\,\,\,\,\,\,\,\,\, + Dyspnea*0.738142233251846 \hfill \\\,\,\,\,\,\,\,\,\,\,\,\,\,\,\,\,\,\,\,\,\,\,\,\,\,\,\,\,\,\,\,\,\,\,\,\,\,\,\,\,\,\, + ESR*2.2996591207573114 + NT \hfill \\\,\,\,\,\,\,\,\,\,\,\,\,\,\,\,\,\,\,\,\,\,\,\,\,\,\,\,\,\,\,\,\,\,\,\,\,\,\,\,\,\, - ProBNP*9.99940698692127 - 2.677061890811556 \hfill \\ \end{gathered}$$

$$\begin{gathered} RS\_combined{\text{ }}model = Clinical{\text{ }}Progress*1.3708223176120489 \hfill \\\,\,\,\,\,\,\,\,\,\,\,\,\,\,\,\,\,\,\,\,\,\,\,\,\,\,\,\,\,\,\,\,\,\,\,\,\,\,\,\,\,\,\,\,\,\,\, + Dyspnea*0.34010196734746717 \hfill \\\,\,\,\,\,\,\,\,\,\,\,\,\,\,\,\,\,\,\,\,\,\,\,\,\,\,\,\,\,\,\,\,\,\,\,\,\,\,\,\,\,\,\,\,\,\,\, + ESR*4.376611992130843 \hfill \\\,\,\,\,\,\,\,\,\,\,\,\,\,\,\,\,\,\,\,\,\,\,\,\,\,\,\,\,\,\,\,\,\,\,\,\,\,\,\,\,\,\,\,\,\,\,\, + NT\_ProBNP*22.35575781100287 \hfill \\\,\,\,\,\,\,\,\,\,\,\,\,\,\,\,\,\,\,\,\,\,\,\,\,\,\,\,\,\,\,\,\,\,\,\,\,\,\,\,\,\,\,\,\,\,\,\, + RS\_Rad*2.873953962898372 \hfill \\\,\,\,\,\,\,\,\,\,\,\,\,\,\,\,\,\,\,\,\,\,\,\,\,\,\,\,\,\,\,\,\,\,\,\,\,\,\,\,\,\,\,\,\,\,\,\,\, - 1.6292291257104095 \hfill \\ \end{gathered}$$

### Models comparison

**Fig. 5:**
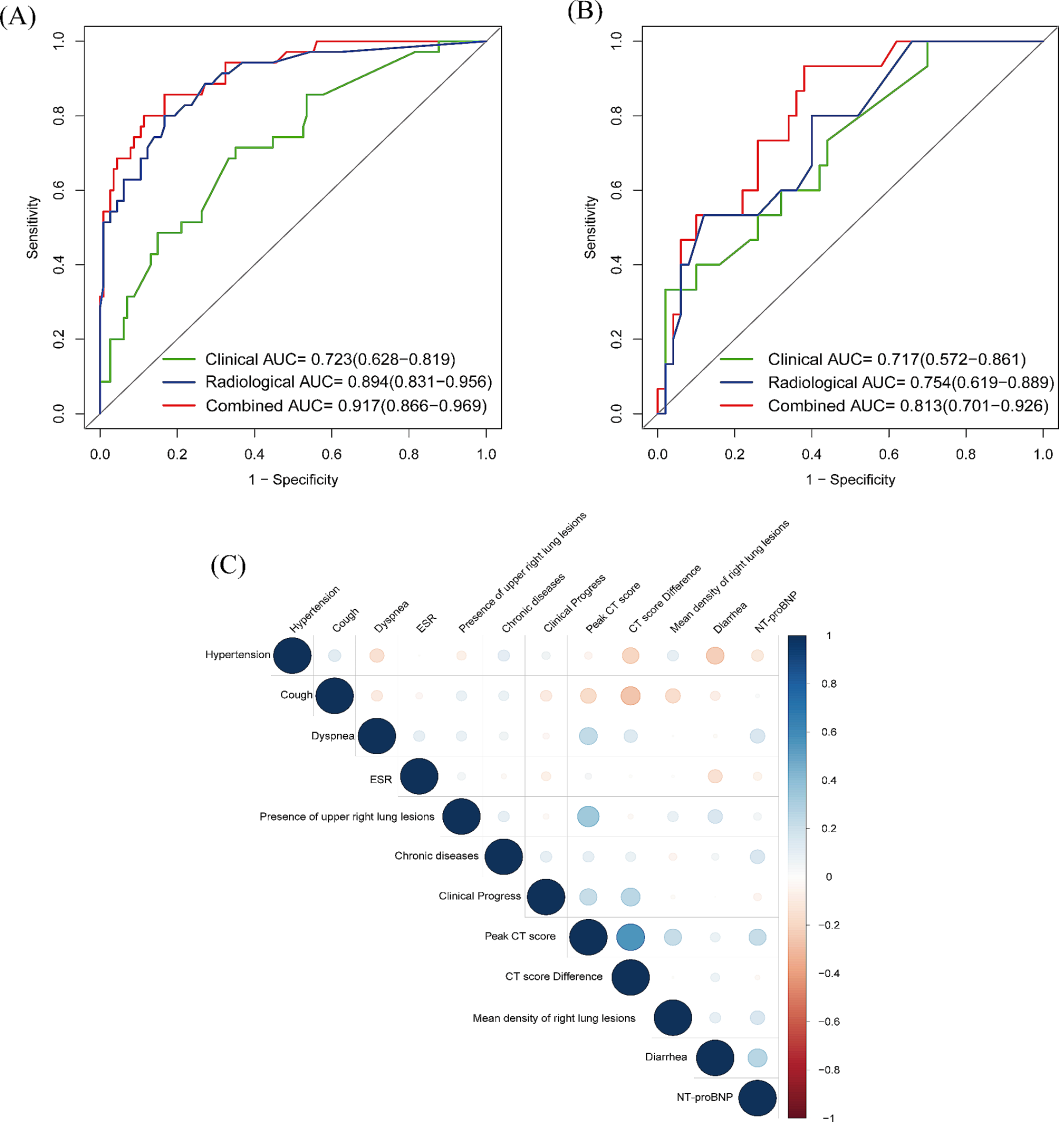
(**A**, **B**) Comparison of ROC curves for assessing the progression of COVID-19 pneumonia in the training group and validation group. (**C**) The correlation coefficient heatmap for clinical variables and radiological features. The larger the value or the darker the color is, the stronger the correlation is

**Fig. 6:**
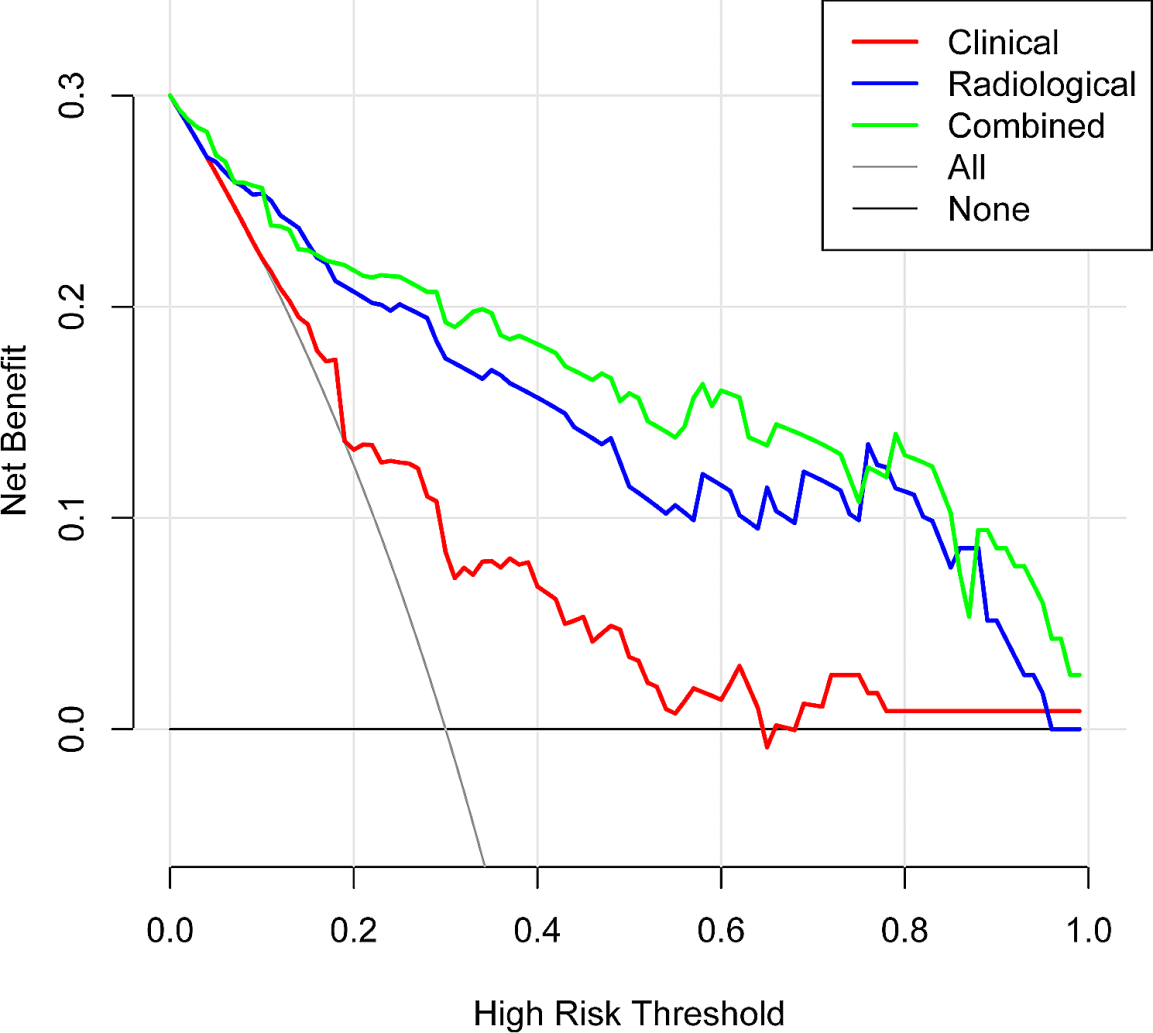
Decision curve analysis (DCA) of the three prediction models. The net benefit curves for the three predictive models are shown. The X-axis indicates the threshold probability of continued progression of pneumonia, and the Y-axis indicates the net benefit

In the training group, the radiological model, clinical model, and combined model demonstrated AUC values of 0.89 (95% CI: 0.83–0.96), 0.72 (95% CI: 0.63–0.82), and 0.92 (95% CI: 0.87–0.97), respectively. For the validation group, these models yielded corresponding AUC values of 0.75 (95% CI: 0.62–0.89), 0.72 (95% CI: 0.57–0.86), and 0.81 (95% CI: 0.70–0.93), respectively. These results showed that the combined model had a higher AUC than the other two in the training and validation groups (Fig. 5). The results of the DCA demonstrated that the combined model outperformed the other two models in terms of clinical benefits (Fig. 6).

## Discussion

The global pandemic has put an insufferable strain on the global healthcare industry, suggesting importance of reliable diagnostic utilities, effective monitoring of disease progression, and individualized therapeutic interventions. The passage or delayed recovery of pneumonia is often due to various factors, including inappropriate initial treatment, co-infection or non-infectious causes, or an abnormal immune response. These complications often lead to extended hospital stays and increased treatment costs and are typically associated with a higher mortality rate [16, 19, 20]. The results of this study demonstrated that integrating quantitative CT features obtained through deep learning automatic segmentation with CT visual features and clinical characteristics effectively enabled the prediction of COVID-19 pneumonia radiological progression in Omicron patients from 2 weeks to 1-month post-admission. With a strong performance in both the training and validation groups, this model poises a high clinical applicability for improving patient prognosis.

For the quantitative analysis of chest CT lesions, the CCTS system displays features of high specificity, rapid interpretation time, and the strongest correlation with lung CT severity [12]. Our study utilized the XGBoost model to select the CT features of lung and COVID-19 pneumonia. Previous research has demonstrated that XGBoost outperforms the logistic regression functions and random forest in predicting critical cases of COVID-19 pneumonia [21]. The four CT features that make up the radiological model in this study included the CT score difference, mean density of right lung lesions, presence of upper right lung lesions, and peak CT score. The SHAP summary plot revealed that the CT score difference emerged as the most influential feature in the model, exhibiting a positive correlation with the predicted outcome. The CT score difference represents the severity of the pneumonia; severe progressing COVID-19 pneumonia can involve lung parenchyma more extensively than mild resolving pneumonia. In patients with progressive disease, the inflammatory cascade disrupts the alveolar-vascular basement membrane, leading to diffuse alveolar damage or acute fibrinous organizing pneumonia, which radiologically manifests as extensive parenchymal opacifications and consolidation from the proliferative phase to the fibrotic phase, leading to potentially fatal and irreversible COVID-19 pneumonia [22]. Studies have shown that COVID-19 patients with severe pneumonia have higher peak CT scores during the disease than patients without severe pneumonia, and a pulmonary involvement of > 50% imposes a multiplicative effect on the risk of mortality [23, 24]. Thus, patients with a poor prognosis tend to have higher peak CT scores.

NT-proBNP, ESR, clinical progression, and dyspnea are risk factors for the continued progression of pneumonia in patients. Bruns et al. demonstrated that dyspnea upon admission was independently associated with delayed radiographic resolution on day 10 of pneumonia [25]. Symptoms of respiratory distress serve as risk factors for a poor prognosis and increase the likelihood of progressing to critical illness or even death [26]. Despite good viral control, the potential for immune response and lung injury continues to advance during severe COVID-19 [27]. Dyspnea indicates impaired lung function and hypoxia, thus warranting vigilance for further deterioration of the patient’s condition upon its occurrence. The combination of inflammation and increased lung permeability due to inspiratory negative intrathoracic pressure leads to interstitial lung edema [28]. Once interstitial lung edema reaches a certain level, the lung’s gas volume decreases, reducing tidal volume at a given inspiratory pressure, causing respiratory distress and further lesion progression [29]. Elevated levels of NT-proBNP serve as a potential indicator of impaired cardiac functionality or heart failure. High ESR results from stronger inflammatory responses and increased protein expression in the acute phase of severe COVID-19 [30]. Previous studies have shown that NT-proBNP and ESR are independent prognostic factors affecting the severity and mortality of COVID-19 patients [31, 32].

Zhou et al. showed that the evolution of COVID-19 pneumonia included an early rapid progression period (1–7 days), a late peak period (8–14 days), and an absorption period (> 14 days) [33]. A previous study showed that the pattern of progression of COVID-19 pneumonia that peaked after 14 days was often relatively static on CT, with these patients having low CT scores throughout the follow-up period and a very mild clinical course of disease [34]. This is different from our findings, possibly due to the small sample size of patients with this pattern of pneumonia progression in previous studies, with a younger average age and less severe initial symptoms. In contrast, our patients were hospitalized and generally older with more underlying diseases.

There are some limitations to the study. Firstly, the limited sample size and absence of an external validation cohort restrict the applicability of our findings. Second, as the disease advances, the size of the lesion may expand. At the same time, the density reduces, marking the recovery phase, which could result in overestimating the CT score. Additionally, patients could acquire other bacterial or viral infections concurrently during a COVID-19 infection. Only COVID-19 pneumonia data were included in our study, and there is a lack of controlled data or assessments for other pneumonia types. Future studies are needed to assess the robustness and applicability of our model across different types of pneumonia and different data sets. Lastly, this study did not incorporate factors related to different treatment approaches.

## Conclusion

In conclusion, our study developed a combined radiological-clinical model that effectively predicted the individualized progression of COVID-19 pneumonia from 2 weeks to 1-month post-admission. Deep learning for automatic segmentation of quantitative CT features has reduced the time and staffing costs associated with this combined model, enhancing its practical value in clinical settings. Our model may be helpful in tailoring precise therapeutic strategies and can serve as an early warning tool for patients at a high risk of disease progression, potentially improving overall patient prognosis and paving the way for more effective strategies in both current and future outbreaks.

### Electronic supplementary material

Below is the link to the electronic supplementary material.

Supplementary Material 1

## Data Availability

The datasets generated and analyzed during the current study are not publicly available but are available from the corresponding author on reasonable request.

## Abbreviations

AI: Artificial intelligence
AIC: Akaike Information Criterion
AST: Aspartate aminotransferase
CCTS: Chest CT scoring
COVID-19: Corona virus disease 2019
CRP: C-reactive protein
CT: Chest computed tomography
DCA: Decision curve analysis
ESR: Erythrocyte sedimentation rate
ICU: Intensive care unit
IL-6: Interleukin-6
LASSO: Least Absolute Shrinkage and Selection Operator
LDH: Lactic dehydrogenase
NT-proBNP: N-terminal prohormone of brain natriuretic peptide
ROC: Receiver Operating Characteristic
RS: Risk scores
SARS-CoV-2: Severe acute respiratory syndrome coronavirus 2
SD: Standard deviation
SHAP: Shapley’s Additive interpretation
TNI: Troponin I
XGBoost: eXtreme Gradient Boosting
