# Supplementary material for: Prediction of short-term progression of COVID-19 pneumonia based on chest CT artificial intelligence: during the Omicron epidemic
Source: BMC Infect Dis. 2024 Jun 17;24:595. doi: 10.1186/s12879-024-09504-9 (PMC11181585; doi:10.1186/s12879-024-09504-9)
Supplement: Supplementary file 1 — Supplementary Material 1 [file 12879_2024_9504_MOESM1_ESM.docx]

**Supp_Table.** Clinical information of patients in training and validation cohorts

| **Characteristics** | **Overall** | **Training Group** | **Validation Group** | **P-Value** |
| --- | --- | --- | --- | --- |
| n | 214 | 149 | 65 |  |
| Age, mean (SD) | 74.3 (13.7) | 73.6 (13.9) | 76.0 (13.1) | 0.234 |
| Gender, n (%) |  |  |  | 0.897 |
| Female | 76 (35.5) | 52 (34.9) | 24 (36.9) |  |
| Male | 138 (64.5) | 97 (65.1) | 41 (63.1) |  |
| Initial Clinical Grading, n (%) |  |  |  | 0.133 |
| Non-serious | 117 (54.7) | 87 (58.4) | 30 (46.2) |  |
| Serious | 97 (45.3) | 62 (41.6) | 35 (53.8) |  |
| Worst Clinical Grading, n (%) |  |  |  | 0.376 |
| Non-serious | 97 (45.3) | 71 (47.7) | 26 (40.0) |  |
| Serious | 117 (54.7) | 78 (52.3) | 39 (60.0) |  |
| Clinical Progress, n (%) |  |  |  | 1.000 |
| No | 183 (85.5) | 127 (85.2) | 56 (86.2) |  |
| Yes | 31 (14.5) | 22 (14.8) | 9 (13.8) |  |
| Hypertension, n (%) |  |  |  | 0.988 |
| No | 94 (43.9) | 66 (44.3) | 28 (43.1) |  |
| Yes | 120 (56.1) | 83 (55.7) | 37 (56.9) |  |
| Diabetes, n (%) |  |  |  | 0.214 |
| No | 143 (66.8) | 104 (69.8) | 39 (60.0) |  |
| Yes | 71 (33.2) | 45 (30.2) | 26 (40.0) |  |
| Cardiovascular diseases, n (%) |  |  |  | 1.000 |
| No | 132 (61.7) | 92 (61.7) | 40 (61.5) |  |
| Yes | 82 (38.3) | 57 (38.3) | 25 (38.5) |  |
| Respiratory disease, n (%) |  |  |  | 1.000 |
| No | 191 (89.3) | 133 (89.3) | 58 (89.2) |  |
| Yes | 23 (10.7) | 16 (10.7) | 7 (10.8) |  |
| Hematological disorders, n (%) |  |  |  | 0.233 |
| No | 191 (89.3) | 130 (87.2) | 61 (93.8) |  |
| Yes | 23 (10.7) | 19 (12.8) | 4 (6.2) |  |
| Chronic diseases, n (%) |  |  |  | 0.804 |
| No | 174 (81.3) | 120 (80.5) | 54 (83.1) |  |
| Yes | 40 (18.7) | 29 (19.5) | 11 (16.9) |  |
| Surgical trauma, n (%) |  |  |  | 0.970 |
| No | 114 (53.3) | 80 (53.7) | 34 (52.3) |  |
| Yes | 100 (46.7) | 69 (46.3) | 31 (47.7) |  |
| Fever, n (%) |  |  |  | 0.737 |
| No | 34 (15.9) | 25 (16.8) | 9 (13.8) |  |
| Yes | 180 (84.1) | 124 (83.2) | 56 (86.2) |  |
| Cough, n (%) |  |  |  | 0.620 |
| No | 37 (17.3) | 24 (16.1) | 13 (20.0) |  |
| Yes | 177 (82.7) | 125 (83.9) | 52 (80.0) |  |
| Diarrhea, n (%) |  |  |  | 0.104 |
| No | 196 (91.6) | 140 (94.0) | 56 (86.2) |  |
| Yes | 18 (8.4) | 9 (6.0) | 9 (13.8) |  |
| Vomit, n (%) |  |  |  | 0.276 |
| No | 196 (91.6) | 139 (93.3) | 57 (87.7) |  |
| Yes | 18 (8.4) | 10 (6.7) | 8 (12.3) |  |
| Dyspnea, n (%) |  |  |  | 0.335 |
| No | 137 (64.0) | 99 (66.4) | 38 (58.5) |  |
| Yes | 77 (36.0) | 50 (33.6) | 27 (41.5) |  |
| CRP, mean (SD) | 73.4 (62.5) | 71.5 (61.5) | 77.7 (64.9) | 0.516 |
| WBC, mean (SD) | 7.2 (9.2) | 6.6 (6.2) | 8.7 (13.8) | 0.237 |
| Lymphocyte count, mean (SD) | 1.7 (8.5) | 1.4 (4.8) | 2.5 (13.7) | 0.506 |
| Lymphocyte percentage, mean (SD) | 18.5 (15.9) | 19.5 (16.4) | 16.1 (14.6) | 0.136 |
| Neutrophil count, mean (SD) | 5.0 (3.4) | 4.7 (3.2) | 5.6 (3.8) | 0.123 |
| Neutrophil percentage, mean (SD) | 72.2 (18.6) | 71.4 (18.5) | 74.1 (18.8) | 0.336 |
| PCT, mean (SD) | 1.9 (7.3) | 2.1 (7.9) | 1.6 (5.7) | 0.656 |
| ESR, mean (SD) | 33.9 (24.9) | 33.7 (23.3) | 34.3 (28.0) | 0.915 |
| D-Dimer, mean (SD) | 12.2 (130.9) | 15.3 (156.6) | 5.2 (20.1) | 0.446 |
| AST, mean (SD) | 37.8 (31.1) | 36.7 (26.7) | 40.4 (39.4) | 0.498 |
| Ferritin, mean (SD) | 1098.1 (1765.2) | 1104.0 (1955.0) | 1084.3 (1243.0) | 0.939 |
| LDH, mean (SD) | 292.9 (166.0) | 287.9 (134.6) | 303.9 (220.8) | 0.590 |
| TnI, mean (SD) | 1.7 (16.0) | 1.8 (18.8) | 1.5 (6.6) | 0.871 |
| NT-ProBNP, mean (SD) | 1935.0 (5103.6) | 2176.2 (6008.9) | 1349.4 (1410.9) | 0.456 |
| IL-6, mean (SD) | 221.1 (991.9) | 190.1 (983.9) | 298.8 (1020.3) | 0.567 |

SD, standard deviation;
